# Design and Preparation of Polysulfide Flexible Polymers Based on Cottonseed Oil and Its Derivatives

**DOI:** 10.3390/polym12091858

**Published:** 2020-08-19

**Authors:** Yurong Chen, Yanxia Liu, Yidan Chen, Yagang Zhang, Xingjie Zan

**Affiliations:** 1 School of Materials and Energy, University of Electronic Science and Technology of China, Chengdu 611731, China; chenyurong@cms.net.cn (Y.C.); liuyanxia@ms.xjb.ac.cn (Y.L.); 2 Department of chemical and environmental engineering, Xinjiang Institute of Engineering, Urumqi 830026, China; chenyd2020@ms.xjb.ac.cn; 3 Xinjiang Technical Institute of Physics and Chemistry, Chinese Academy of Sciences, Urumqi 830011, China; zanxj@ms.xjb.ac.cn

**Keywords:** polysulfide-derived polymers, cottonseed oil, fatty acid of cottonseed oil, sodium soap of cottonseed oil

## Abstract

Polysulfide-derived polymers with a controllable density and mechanical strength were designed and prepared successfully using bio-based cottonseed oil (CO) and its derivatives, including fatty acid of cottonseed oil (COF) and sodium soap of cottonseed oil (COS). The reaction features of CO, COF and COS for polysulfide polymers were investigated and compared. Based on the free radical addition mechanism, COF reacts with sulfur to generate serials of polysulfide-derived polymers. COF strongly influences the density and tensile strength of these polymer composites. Whereas COS was not involved in the reaction with sulfur, as a filler, it could increase the density and tensile strength of polysulfide-derived polymers. Moreover, the results showed that these samples had an excellent reprocessability and recyclability. These polysulfide-based polymers, with an adjustable density and mechanical strength based on CO and derivatives, could have potential applications as bio-based functional supplementary additives.

## 1. Introduction

Elemental sulfur derives mainly from industrial process such as petroleum refining. It is currently recognized as a vital basic chemical stock for rubber production, chemical fertilizer, antimicrobial agents and chemical dyes [1,2,3,4,5]. Although elemental sulfur has been applied in many fields, it is extensively stockpiled every year because production far surpasses demand [6]. Since 1,3-diisopropenyl benzene was found to stabilize polysulfide chains in inverse vulcanization and the resulting thermoplastic copolymers exhibited great processability and promising potential for applications in the electrical industry as cathodes [7], various functional sulfur-containing polymers and sulfur–organic copolymers synthesized by using different cross-linkers have been reported [8,9,10]. These sulfur-containing polymers and sulfur–organic copolymers have drawn considerable attention due to their application potentials for solid electrodes [11,12], camera lenses and medium infrared ranges [13], as well as heavy metal remediation [14]. In order to make the best of mass-produced sulfur and meet the demands of sustainable development, it would be desirable to seek renewable and sustainable bio-based materials to prepare value-added materials with sulfur. Bio-based materials feature many advantages such as their inexpensiveness, accessibility and availability in large quantities. The design and synthesis of bio-based functional materials also fits in with and benefits green chemistry and sustainable engineering, aimed at making the best of natural resources with more environmentally benign and eco-friendly approaches [15,16,17,18,19].

Vegetable oils, such as canola oil, sunflower oil and linseed oil [20], have been explored for the preparation of functional sulfur–organic copolymers with the method of inverse vulcanization. Cotton, as a vital kind of commercial crop, has a wide range of planting areas worldwide and a higher yield compared with other commercial crops. Therefore, cottonseed oil has more advantages in view of yield and price than other vegetable oils. Currently, the explorations for cottonseed oil are concentrated on the development of biodiesel with lower environmental pollution, lower production costs and greater safety [21,22,23,24], whereas polysulfide-derived polymers based on cottonseed oil and derivatives are rarely reported on. It is highly desirable to design and prepare value-added functional polymer composites with cottonseed oil and its derivatives.

In our previous work [25], cottonseed oil was used as a renewable cross-linker to react with industrial byproduct sulfur and the resulting sulfur-containing plant rubber polymers were prepared successfully. These plant rubber polymers could remove mercury ions from aqueous solution as bio-based absorbents. Traditional rubber materials could serve as supplementary additives for improving the mechanical properties or densities of polymers [26,27,28,29]. It would be interesting and ideal if the mechanical property and rigidity of these sulfur-containing plant rubber polymers were tunable. In the work reported here, we explored the possibility of preparing value-added sulfur-containing functional polymer with cottonseed oil and its derivatives with goal of achieving controllable densities and adjustable mechanical strength.

In this work, though the inverse vulcanization process, mass-produced sulfur, renewable cottonseed oil (CO) and its derivatives were taken advantage of, and a series of novel polysulfide-based polymers with controllable densities and adjustable mechanical strength were successfully prepared. Cottonseed oil derivatives involved in this work included fatty acid of cottonseed oil (COF) and sodium soap of cottonseed oil (COS). These polysulfide-derived polymers could have potential applications as bio-based functional supplementary additives.

## 2. Materials and Methods

### 2.1. Materials

Cottonseed oil (CO, food grade), fatty acid of cottonseed oil (COF) and sodium soap of cottonseed oil (COS) from cottonseed oil were obtained from Shihezi Kanglong Oil Industry and Trade Company (Shihezi, China). Sulfur (S, powder, ≥99.5%,) was purchased from Tianjin Baishi Chemical Industry Co. Ltd. (Tianjin, China). CO, COF, COS and their mixture, including CO/COF, CO/COS, as well as CO/COF/COS, is referred to as COX collectively for ease of subsequent discussion. Moreover, except for CO, residual raw materials were also called COY in order to differentiate them from COX.

### 2.2. Preparation of Polysulfide-Derived Polymers

About 10.0 g of elemental sulfur powder (S) was added into a 100 mL vial equipped with a magnetic stir bar and then melted while stirring at 150 °C. A certain amount of COX was then added to the above molten liquid, while stirring and heating were continued to ensure efficient mixing and reactions between reactants. After a certain period, the mixture was cooled to room temperature and then the resulting polysulfide-derived polymers were obtained. The related details can be found in Table 1.

### 2.3. Characterization

The morphological analysis of the prepared samples was performed by a field emission scanning electron microscopy (FE-SEM, SUPRA 55vp, ZEISS, Oberkochen, Germany) with an Oxford detector, operating with 2.00 kV electron beams. Infrared (IR) spectra were recorded on a Fourier Transform Infrared Spectrometer (VERTEX-70, Bruker, Karlsruhe, Germany) using the ATR (Attenuated Total Reflectance) method with a wave number ranging from 400 to 4000 cm^−1^. Gas Chromatography–Mass Spectrometry (GC–MS) was acquired on a Headspace injection gas chromatograph mass spectrometer (7890B GC/7697A/5977B MSD, KEYSIGHT, Santa Rosa, CA, America) using a Rtx-5MS column (30 m long × 0.25 mm thickness × 0.25 μm ID), with an injection temperature of 130 °C, column temperature of 270 °C, gas flow rate of 24.0 mL·min^−1^, and electron ionization used to obtained nominal masses. Density analysis was carried out on an electronic densitometer (XF-120MD, Xiongfa, Xiamen, China) with testing samples tailored in the cubic dimension of 10 mm × 10 mm × 10 mm. Mechanical testing was carried out on a microcomputer-controlled electronic tensile testing machine (C43-104, MTS, Rochester, MN, America) according to the national standard GB/T 528-2009 with dumbbell-shaped splines at an elongation rate of 500 mm·min^−1^ and the test length and thickness of splines were 20.0 ± 0.5 mm and 2.0 ± 0.2 mm, respectively.

### 2.4. Self-Healing Experiments

To study the reprocessability and recyclability of the related samples, self-healing experiments were performed by cutting samples into pieces and putting the fragments together for remolding under 10 MPa at 160 °C for 10 min using a thermo-compressor (R3212, Qien, Zhengzhou, China). The ratio of the tensile strength of the healed samples to those of the original one was determined as the recovery ratio to measure the reprocessability and recyclability of samples.

## 3. Results and Discussion

### 3.1. Synthesis and Characterization

**Figure 1:**
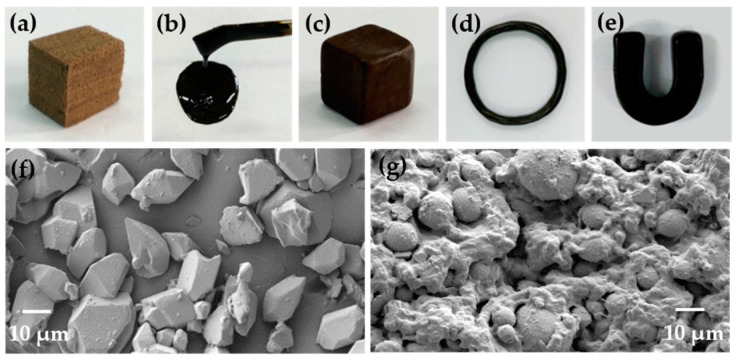
The surface morphology and plasticity of real samples. (**a**,**b**) Digital photos of sulfur cottonseed oil (SCO) and sulfur fatty acid of cottonseed oil (SCOF), respectively; (**c**–**e**) digital images of sulfur–sodium soap of cottonseed oil (SCOS): (**c**) cubic SCOS, (**d**) ring-shaped SCOS and (**e**) U-shaped SCOS; (**f**,**g**) SEM images of SCO and SCOS, respectively (m_S_: m_COX_ = 1:1).

**Figure 2:**
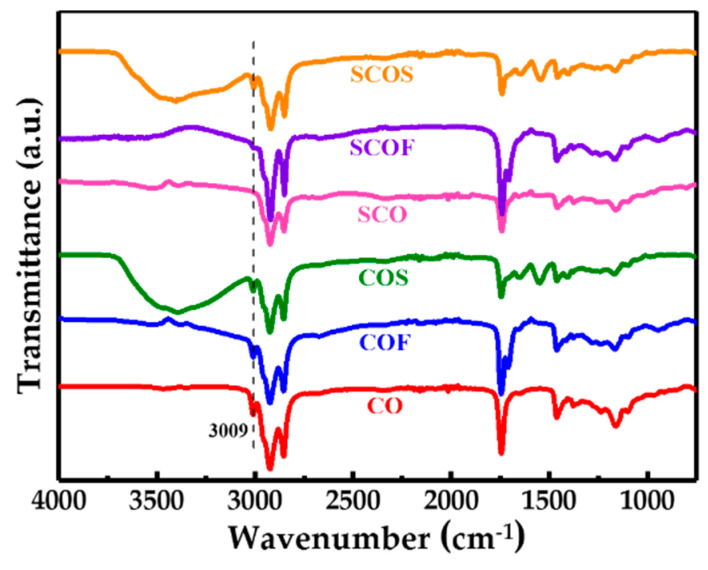
FT-IR spectrogram of the related samples (m_S_: m_COX_ = 1:1).

To investigate whether elemental sulfur could react effectively with cottonseed oil derivatives including fatty acid of cottonseed oil (COF) and sodium soap of cottonseed oil (COS), the resulting polysulfide-derived polymers were analyzed. As depicted in Figure 1a–e, when elemental sulfur reacted with CO, COF and COS, respectively, the resulting polysulfide-derived polymers SCO and SCOS appeared as brown elastic bulk and tanned plastic bulk, respectively, while SCOF appeared as a black viscous fluid. Moreover, the tanned plastic SCOS bulk could also be kneaded into a ring-shaped or U-shaped object at room temperature, which implies that SCOS is quite flexible and features excellent processability compared with SCO and SCOF. Figure 1f,g displays the surface morphology of SCO and SCOS under a scanning electron microscope. There were large numbers of fragments on the surface of SCO, whereas the surface of SCOS was occupied by a sticky object conjoined with many small particles. Furthermore, an FT-IR spectrogram (Figure 2) was also used to analyze the structural changes in SCO and SCOF, as well as SCOS. Compared with raw materials CO, COF and COS, the prepared SCO and SCOF both showed that the moderate-strength peak at 3009 cm^−1^ disappeared (with the exception of SCOS). The absence of the peak was attributed to the vanishing of =C–H bond stretching vibration in cottonseed oil and fatty acid of cottonseed oil [4]. Meanwhile, according to Table 2, the relative intensity of the peak at 3009 cm^−1^ in SCO and SCOF samples decreased by about 90% compared with that of raw materials CO and COF, whereas relative intensity of the peak at 3009 cm^−1^ in SCOS samples had no obvious changes compared with that of the raw material COS. The quantitative analysis of the FT-IR spectrogram further indicated that there existed an obvious reaction between raw materials CO and COF and products SCO and SCOF. The macroscopic and microscopic differences between samples as well the as differences in the FT-IR spectrogram implied differences in chemical reactivity between sulfur and different COX. According to the previous work [20], under a high temperature, elemental sulfur could initiate ring-opening polymerization and further react with the unsaturated double bonds in plant oils and their derivatives. The essence of the reaction was that, above the melting point temperature, 120 °C, sulfur was first melted and then heated up further to a higher temperature to generate sulfur free radicals to further react with C=C bonds in plant oils by the free radical addition mechanism.

**Figure 3:**
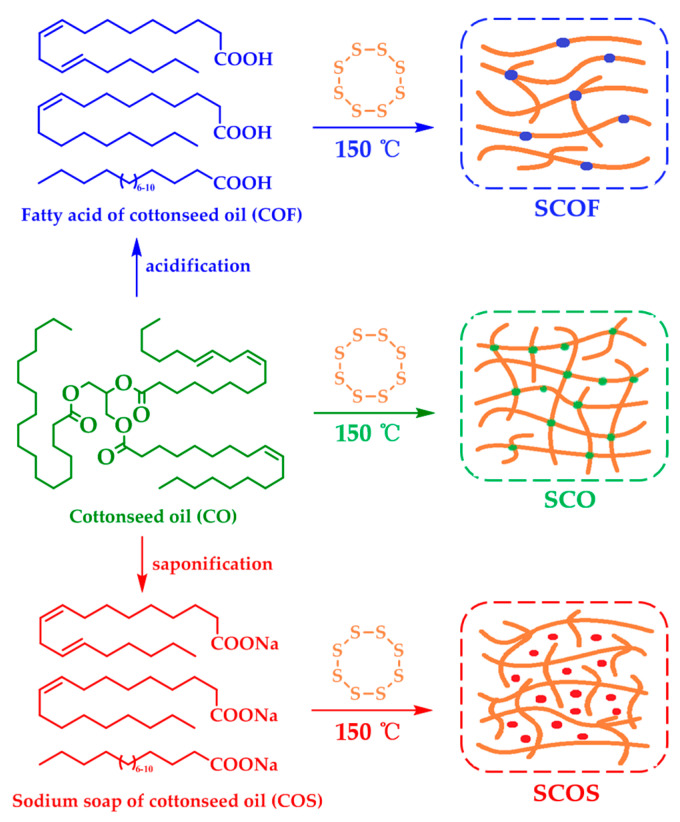
Procedures for the preparation of SCO, SCOF and SCOS, respectively.

GC–MS analysis data (Table 3) show that cottonseed oil contains different fatty acid components, including both saturated and unsaturated fatty acids. There are a large number of C=C bonds derived from unsaturated fatty acid, including linoleic acid and oleic acid. These C=C bonds are the key motifs and act as active cross-linking sites with sulfur to generate cross-linking elastic SCO (Figure 3). This also explains why the =C–H bond stretching vibration in the FT-IR spectra of SCO disappeared compared with raw material CO. The results show that the content of unsaturated fatty acid was about 75% in COF. Theoretically, COF could react with sulfur to generate elastic plant rubber in a similar manner to SCO. However, the resulting SCOF was a viscous fluid instead of presenting solid-state elasticity. This could be due to the fact that the vanishing of glycerinum decreased the cross-linking effect of unsaturated fatty acid in COF, and the obtained SCOF had a low cross-linking degree (Figure 3). This could also explain why the FT-IR spectra of SCO and SCOF were similar but the appearance and character of them were different. Sodium soap based on unsaturated fatty acid was the dominant component of COS, which mainly included sodium linoleate and sodium oleate. In theory, COS could react with sulfur to generate an elasticity similar to SCO. However, the resulting SCOS appeared as a plastic bulk. Moreover, compared with the FT-IR spectrogram of raw material COS, the peak at 3009 cm^−1^ still existed in SCOS, which may imply that there was no reaction between sulfur and COS. This was due to the high melting point of sodium linoleate and sodium oleate, which exceeded 190 °C. Therefore, these sodium soaps were not effectively involved in the reaction with sulfur at 150 °C. Instead, they were mingled with polysulfide fragments as granular padding (Figure 3). The observation of the chemical reactivity with different cottonseed oils and their derivatives was consistent with the above analysis of the SEM images of SCOS (Figure 1f,g).

### 3.2. Density Analysis

**Figure 4:**
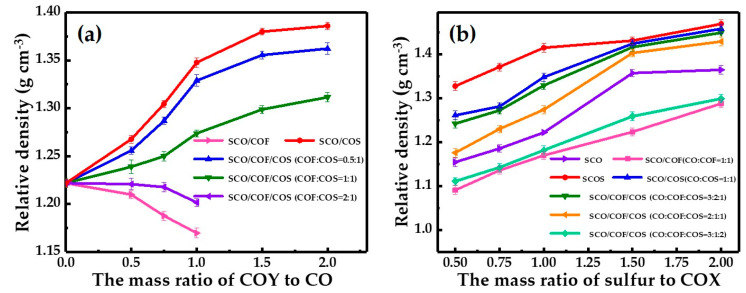
Relative density of the prepared samples. (**a**) Relative density variation in samples versus the mass ratio of COY to CO (m_S_/m_COX_ = 1); (**b**) relative density variation in samples versus the mass ratio of sulfur to CO, COF, COS and their mixture, including CO/COF, CO/COS, as well as CO/COF/COS (COX).

As depicted in Figure 4a, when the mass ratio of sulfur to COX was 1.0, the density of SCO/COF as well as SCO/COF/COS serial samples (while the mass ratio of COF to COS was 2.0) decreased with the increase in the mass ratio of COF or COF/COS to CO and was lower than that of SCO samples. These results imply that the introduction of COF could decrease the density of the prepared polymer composites. On the other hand, the density of SCO/COS and SCO/COF/COS serial samples (while the mass ratio of COF to COS was lower than 2.0) increased significantly with the rising mass ratio of COS or COF/COS to CO and, noticeably, the density of these samples were higher than that of SCO serial samples, which indicated that the introduction of COS could increase the density of the resulting polymer composites. Moreover, Figure 4b reveals that the density of samples gradually increased with the increase in the mass ratio of sulfur to COX, which implies that increasing the sulfur content is helpful for elevating the density of samples to some extent.

### 3.3. Mechanical Strength Analysis

**Figure 5:**
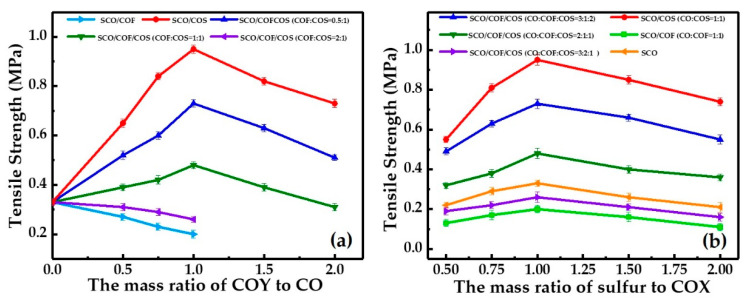
Tensile strength of the prepared samples. (**a**) Tensile strength variation in samples versus the mass ratio of COY to CO (m_S_/m_COX_ = 1); (**b**) tensile strength variation in samples versus the mass ratio of sulfur to COX.

Figure 5a shows that when the mass ratio of sulfur to COX was 1.0, the tensile strength of SCO/COF serial samples decreased with the increasing mass ratio of COF to CO. Similarly, the tensile strength of SCO/COF/COS serial samples decreased with the increasing mass ratio of COF/COS to CO, while the mass ratio of COF to COS was 2.0. Meanwhile, the tensile strength of both SCO/COF and SCO/COF/COS serial samples were lower than that of SCO serial samples, which implies that the introduction of COF could decrease the mechanical strength of samples. However, when the mass ratio of COY to CO was lower than 1.0, the tensile strength of SCO/COS serial samples increased significantly with the increase in the mass ratio of COS to CO. The tensile strength of SCO/COF/COS serial samples also increased with the increasing mass ratio of COF/COS to CO, while the mass ratio of COF to COS was lower than 2.0. Most importantly, the tensile strength of these samples was higher than that of SCO serial samples, which reveals that the introduction of COS could enhance the mechanical strength of samples effectively. However, when the mass ratio of COY to CO was higher than 1.0, the tensile strength of SCO/COS and SCO/COF/COS serial samples (the mass ratio of COF to COS was lower than 2.0) started to decline, which was due to the decreasing cross-linking density resulting from the lessening of the content of CO. Figure 5b demonstrates that the tensile strength of samples initially increased with the increase in the mass ratio of sulfur to COX and gradually reached the maximum when the mass ratio of sulfur to COX was 1.0 and then decreased dramatically. This could be due to local stress concentration on the surface and inside the samples [30] because of the increase in the content of sulfur.

### 3.4. Reprocessability and Recyclability

**Figure 6:**
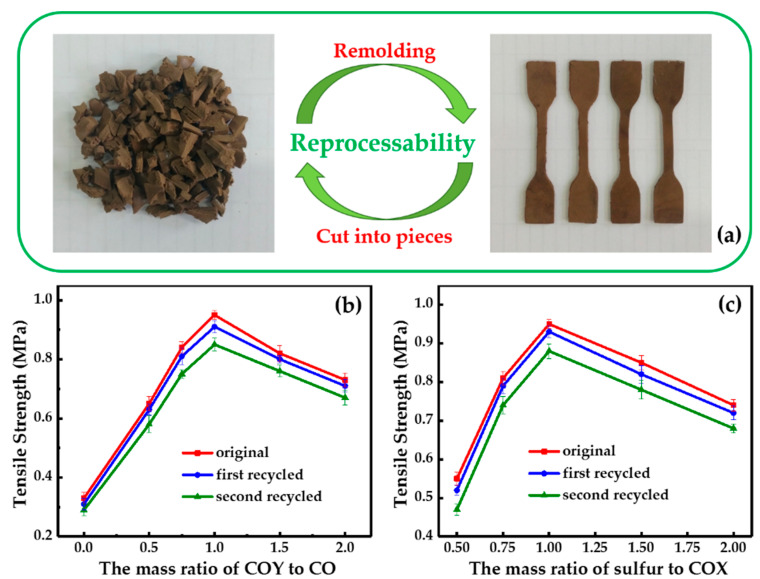
Reprocessability and recyclability of SCO/COS serial samples. (**a**) Digital photos of thermal reprocessing ability of SCO/COS serial samples; (**b**) tensile strength variation in SCO/COS serial samples versus the mass ratio of COY to CO after multiple reprocesses; (**c**) tensile strength variation in SCO/COS serial samples versus the mass ratio of sulfur to COX after multiple reprocesses.

To further study the reprocessability and recyclability of the prepared samples, SCO/COS serial samples with a higher density and tensile strength were chosen as representative samples. As shown in Figure 6a, SCO/COS serial samples can be remolded into coherent and smooth dumbbell-shaped splines when they are cut into small pieces and after hot pressing. Figure 6b,c show the tensile strength variation in SCO/COS serial samples versus the mass ratio of reactants after multiple reprocesses. The tensile strength of SCO/COS serial samples exhibited a recovery ratio above 90% after first reprocessing and the secondary recovery rate was above 85%, which was due to a large number of reversible disulfide bonds in the samples. These results demonstrate that the reversible cross-linking made the samples capable of reprocessing and recycling [31]. However, the results show that the tensile strength of these materials decreased significantly after more than two cycles. Our hypothesis was that the cross-linked sulfur–sulfur bonds would degrade after more than two cycles and simple hot pressing would not be efficient enough to aid in recovering and rebuilding those damaged bonds. These are actually the disadvantages of this type of material and it is important to find a way to solve these problems.

## 4. Conclusions

A series of polysulfide-derived polymers with a controllable density and mechanical strength were prepared successfully based on cottonseed oil (CO) and its derivatives, including fatty acid of cottonseed oil (COF) and sodium soap of cottonseed oil (COS). Based on the free radical addition mechanism, which is similar to the reaction mechanism of SCO, COF reacted with sulfur generates serial samples containing COF. COF can decrease the density and tensile strength of polysulfide-based polymers, whereas COS was not effectively involved in the reaction with sulfur due to the high melting point of sodium linoleate and sodium oleate. Even so, COS could act as a padding component, which could increase the density and tensile strength of polysulfide-derived polymers. The results demonstrated that the prepared polymer composites had an excellent reprocessability and recyclability, attributed to the large number of reversible disulfide bonds formed in the formation of plant rubber. These polysulfide-derived polymers with a controllable density and mechanical strength, based on CO and derivatives, could have potential applications as bio-based functional supplementary additives.

## Figures and Tables

**Table 1:** The preparing conditions of the resulting polysulfide-derived polymers.

Samples	Components	Mass Ratio of Reactants	Temperature (°C)	Time ^1^ (min)
SCO	S:CO	0.5, 0.75, 1, 1.5, 2:1	150	25–30
SCOF	S:COF	0.5, 0.75, 1, 1.5, 2:1	150	25–30
SCOS	S:COS	0.5, 0.75, 1, 1.5, 2:1	150	20–25
SCO/COF	CO:COF	1:0.5, 0.75, 1.0	150	15–20
	S:CO/COF	0.5, 0.75, 1, 1.5, 2:1		
SCO/COS	CO:COS	1:0.5, 0.75, 1, 1.5, 2	150	15–20
	S:CO/COS	0.5, 0.75, 1, 1.5, 2:1		
SCO/COF/COS	COF:COS	0.5, 1, 2:1	150	15–20
	CO:COF/COS	1:0.5, 0.75, 1, 1.5, 2		
	S:CO/COF/COS	0.5, 0.75, 1, 1.5, 2:1		

Time ^1^ refers to the period from the moment that all reactants were fully mixed together to the moment that the reactions were finished, which does not include the period of cooling samples to room temperature.

**Table 2:** Quantitative data for FT-IR spectrogram of the related samples (m_S_: m_COX_ = 1:1).

Samples	Peak (cm^−1^)	Relative Intensity (%)
CO	3009	33.82
COF	3009	45.22
COS	3009	47.81
SCO	3009	2.09
SCOF	3009	3.98
SCOS	3009	47.08

**Table 3:** Representative methyl esterification products of cottonseed oil by Gas Chromatography–Mass Spectrometry (GC–MS) analysis.

Fatty Acid Methyl Ester	Relative Content (%)
Methyl tetradecanoate	0.31
Methyl palmitate	16.70
Methyl stearate	1.45
Methyl oleate	15.25
Methyl linoleate	60.61
